# Congenital left atrial appendage aneurysm: A rare case report and literature review

**DOI:** 10.1097/MD.0000000000009344

**Published:** 2018-01-12

**Authors:** Bin Wang, He Li, Li Zhang, Lin He, Jing Zhang, Cong Liu, Jing Wang, Qing Lv, Xiaoke Shang, Jinping Liu, Mingxing Xie

**Affiliations:** a Department of Ultrasound; b Hubei Key Laboratory of Molecular Imaging.; c Department of Cardiovascular Surgery, Union Hospital, Tongji Medical College, Huazhong University of Science and Technology, Wuhan, China.

**Keywords:** atrial appendage aneurysms, echocardiography, left

## Abstract

**Rationale:**

Left atrial appendage aneurysms (LAAA) are rare. Patients with LAAA are often diagnosed incidentally or after cardiac tachyarrhythmia or systemic thromboembolism happen. Early diagnosis and surgical resection is of utmost importance to prevent hazardous adverse events.

**Patient concerns:**

We present a case of 46-year-old man with congenital LAAA. The individual in this manuscript has given written informed consent to publish these case details.

**Diagnoses:**

Imaging studies, such as echocardiography, cardiovascular computed tomography (CT) and magnetic resonance imaging (MRI), demonstrated the large cavity arising from the left atrial appendage. The diagnosis of LAAA was confirmed.

**Interventions:**

The patient underwent an aneurysmectomy without any complications.

**Outcomes:**

TTE confirmed the disappearance of the LAAA from the left parasternal short-axis view of the aortic root postoperatively. The patient remained asymptomatic without any adverse events at his 3-month follow-up visits.

**Lessons:**

The associated high risk of life-threatening complications and the relative ease of surgical removal suggest that prompt evaluation should be considered in patients with lesions adjacent to the left heart border.

## Abstract 2

Supplemental Digital Content is available in the text

## 1 Introduction

The left atrial appendage aneurysm (LAAA) is extremely rare. It can be caused by congenital dysplasia of the atrial muscles or secondary to mitral valve disease. Most patients are asymptomatic, while palpitations, dyspnea, or chest pain can be found to be main symptoms. Herein, we present the case of a 46-year-old man with congenital LAAA. This study was approved by the local research ethics committee at Union Hospital, Tongji Medical College, Huazhong University of Science and Technology, China. The informed consent was given by the patient in this manuscript. Furthermore, we review LAAA-related literature published from 1962 to 2016 to provide detailed information about its symptoms, diagnosis, treatment, and progress.

## 2 Case report

### 2.1 Case history

**Figure 1:**
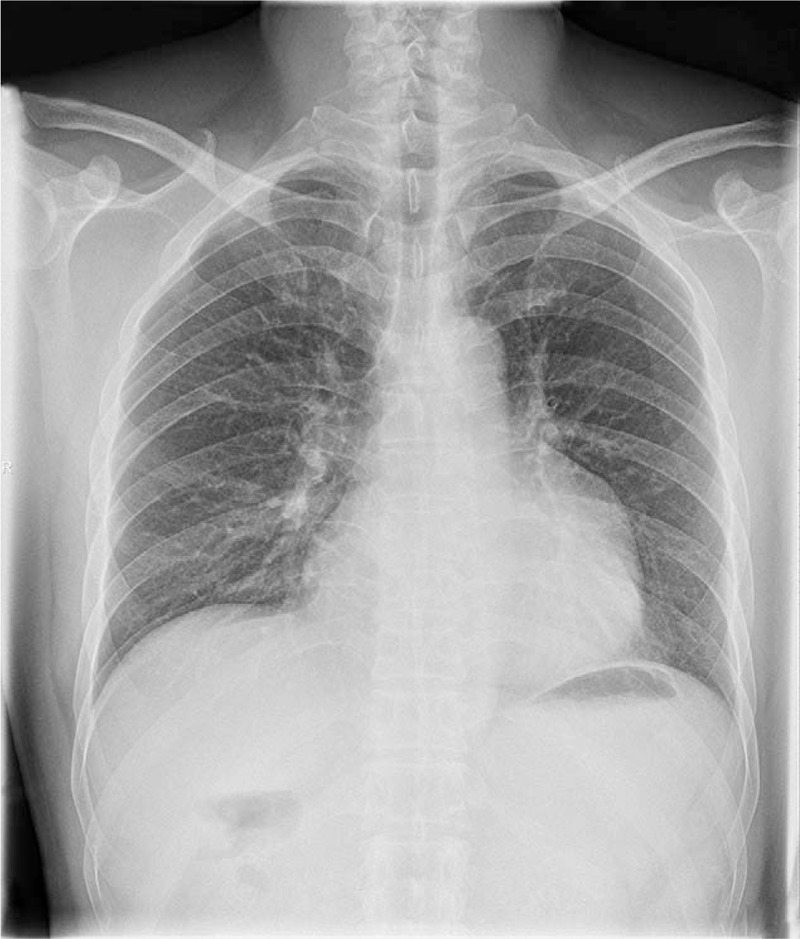
The posteroanterior chest x-ray demonstrating abnormal left heart border.

A 46-year-old man presented to our hospital with chest discomfort for 3 months. He denied history of cardiac disease or surgery. Two-dimensional transthoracic echocardiography (2D-TTE) in a hospital 2 months ago revealed a huge pericardial cyst. Physical examination and laboratory findings were unremarkable. The electrocardiogram, however, showed sinus rhythm with a notched P wave, and the chest x-ray revealed that the left border of the heart was greatly enlarged (Fig. 1).

**Figure 2:**
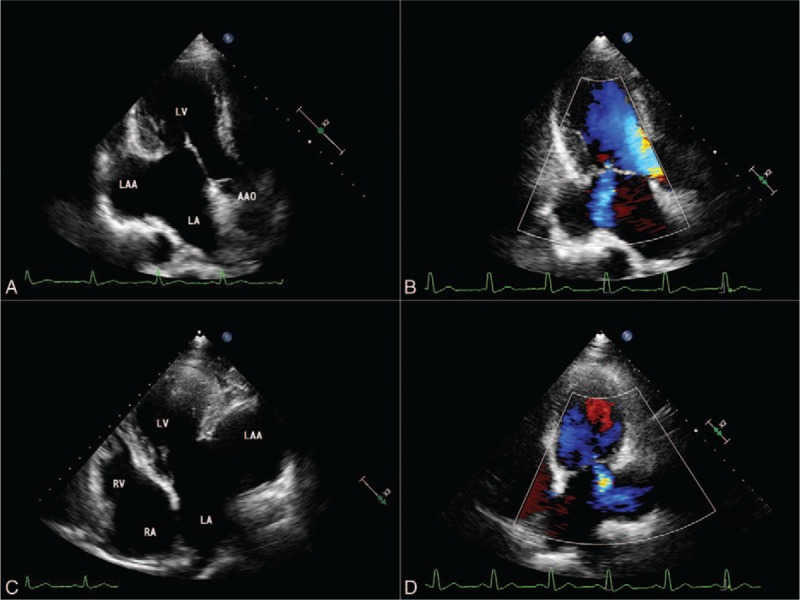
Transthoracic echocardiograms. Apical 3-chamber (A, B) and 4-chamber (C, D) views showing a 8.4 × 6.8 × 4.3-cm size mass communicating to LA and the presence of mild mitral regurgitation. AAO = ascending aorta, LA = left atrium, LAA = left atrial appendage, LV = left ventricle, RA = right atrium, RV = right ventricle.

**Figure 3:**
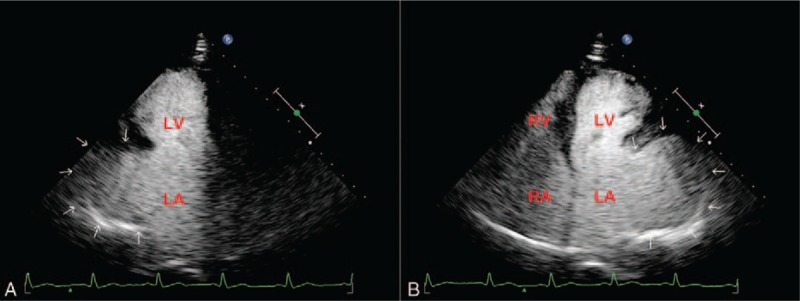
Contrast echocardiograms. Apical 3-chamber (A) and 4-chamber (B) views confirming this mass (arrow) communicating to the LA. LA = left atrium, LV = left ventricle, RA = right atrium, RV = right ventricle.

**Figure 4:**
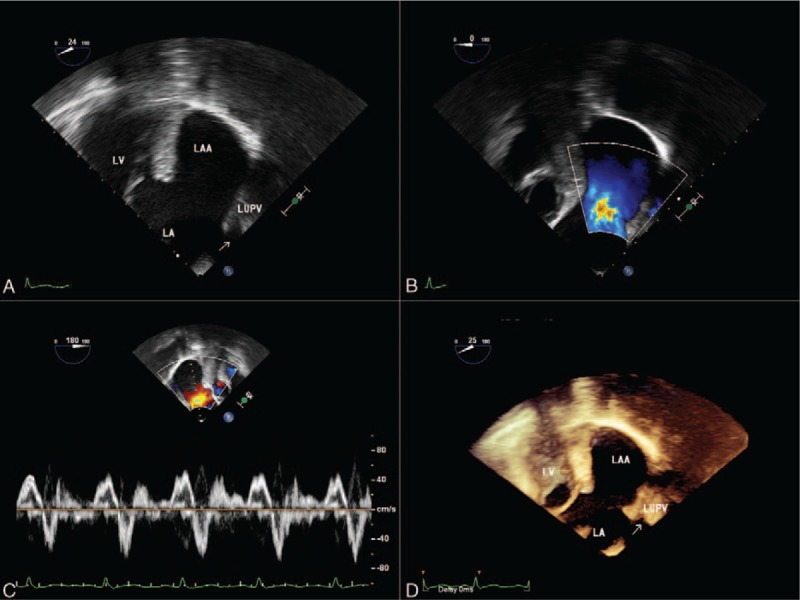
Transesophageal echocardiograms. The mid-esophageal 2-chamber and LAA view (A) revealing a giant LAA. Color Doppler flow imaging (B) showing communication of flow between LAA and the LA. Pulsed wave Doppler (C) echocardiographic examination of blood flow at the orifice. Three-dimensional view (D) confirming the presence of giant LAA. LA = left atrium, LAA = left atrial appendage, LV = left ventricle, LUPV = left upper pulmonary vein.

2D-TTE (Philips IE33; Philips Healthcare, Eindhoven, the Netherlands) in our hospital was suggestive of a large cavity lateral to the heart. The apical 3-chamber and 4-chamber views demonstrated this cavity, measuring 8.4 × 6.8 cm, related to and communicated with the left atrium via a broad neck whose diameter was 4.3 cm (Fig. 2A and C) (supplementary video 1 and supplementary video 3). Left ventricular size and systolic function were normal, with mild mitral regurgitation (Fig. 2B and D) (supplementary video 2 and supplementary video 4). Contrast-enhanced ultrasound findings demonstrated the echo-contrast agent filled of the LAAA (Fig. 3) (supplementary video 5 and supplementary video 6). And transesophageal echocardiography (TEE) and real-time 3-dimensional echocardiogram (RT-3DE) demonstrated this enlarged cavity arising from the LAA with intense spontaneous echo contrast, but without thrombi (Fig. 4A and D) (supplementary video 7 and supplementary video 9). Low velocity blood flow between LA and aneurysm are detected by color Doppler and pulsed Doppler echocardiography (Fig. 4B and C) (supplementary video 8).

### 2.2 Computed tomography and magnetic resonance imaging

**Figure 5:**
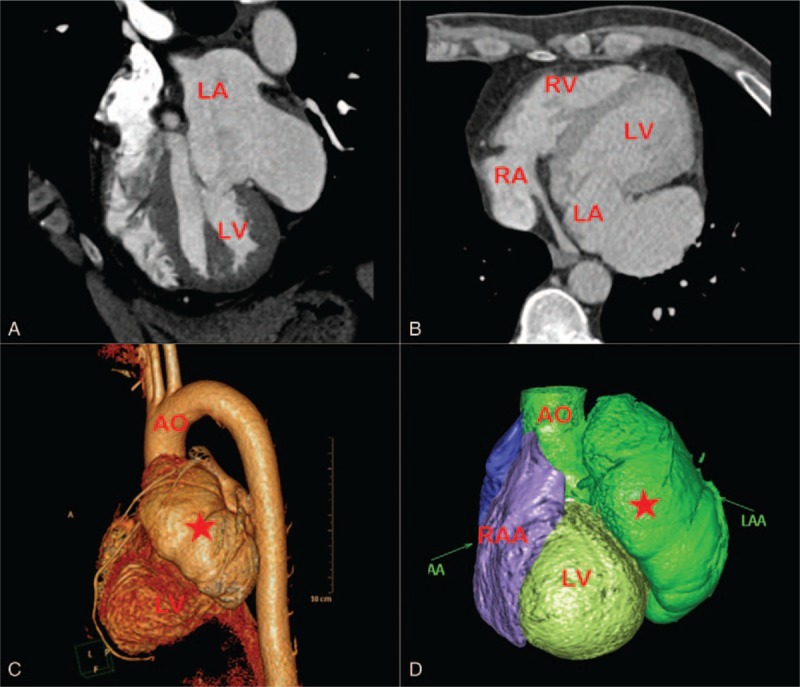
CT views characterizing the giant LAA measuring 9.6 × 4.5 × 3.8 cm. AAO = ascending aorta, LA = left atrium, LAA = left atrial appendage, LV = left ventricle, RA = right atrium, RV = right ventricle, RAA = right atrial appendage.

**Figure 6:**
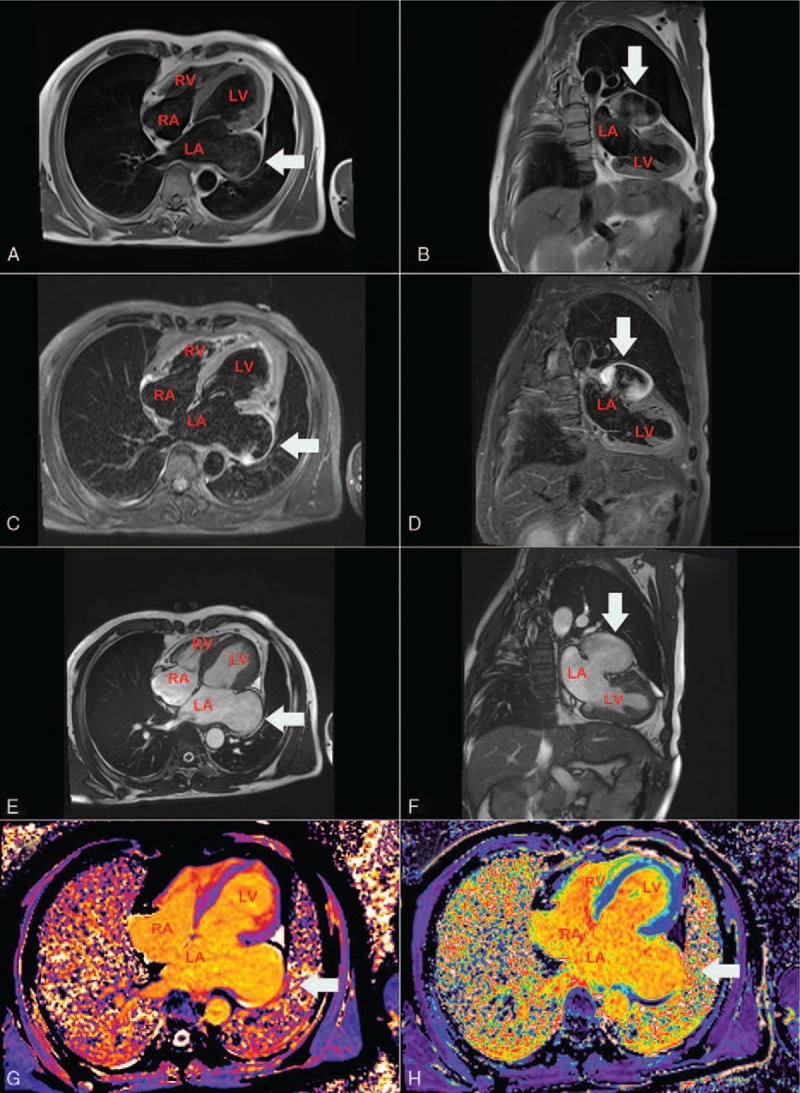
MRI. Axial spin-echo T1-weighted (E) and Coronal spin-echo T1-weighted image (F) showing the LAA aneurysm with no evidence of thrombus. LA = left atrium, LV = left ventricle, MRI = magnetic resonance imaging, RA = right atrium, RV = right ventricle.

To further delineate the anatomy of this large cavity and its surrounding structure, this patient underwent computed tomography (CT) and magnetic resonance imaging (MRI). CT and MRI demonstrated that a 9.6 × 4.5 × 3.8-cm size LAA compressing the left ventricular wall (Figs. 5 and 6). The pericardium was noted to be intact. Therefore, the echocardiography findings were verified, and the diagnosis of LAAA was confirmed.

### 2.3 Surgical and pathological findings

**Figure 7:**
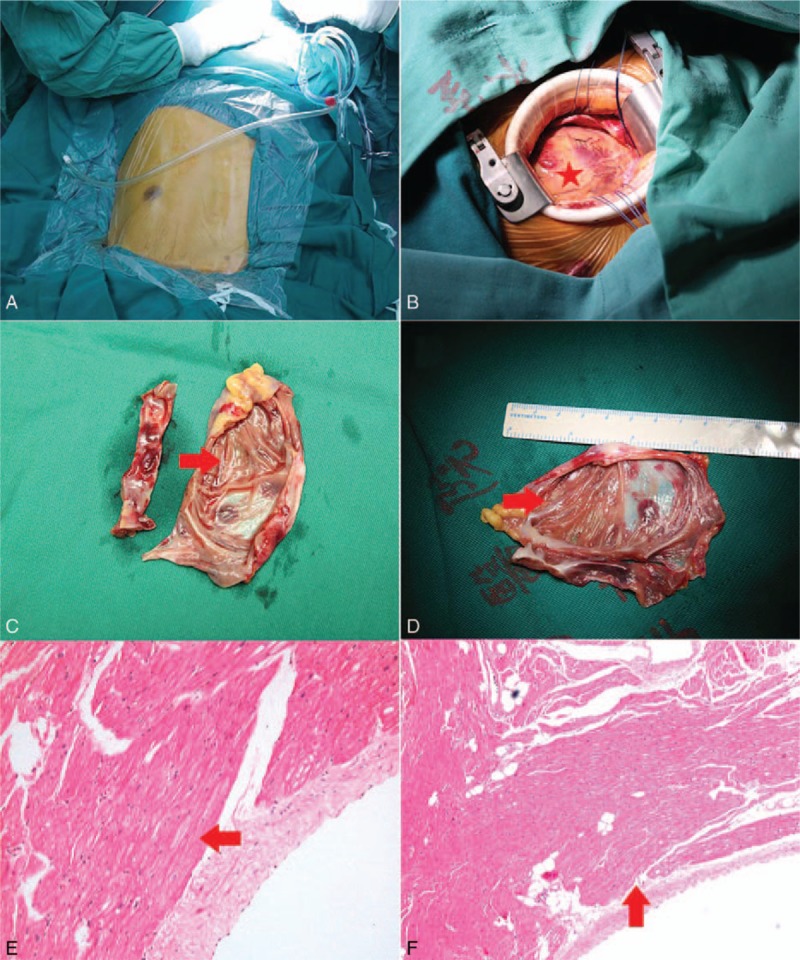
Intraoperative views (A–D) and histopathological pictures (E, F). The resected aneurysm measured 8.7 × 7.0 cm (C, D) through a limited left thoracotomy (A, B). Histopathological section of the aneurysm showing the myocardial thinning with fatty infiltration.

Given the potential risk of thromboembolic events, the patient was suggested to take surgical removal of the aneurysm. After intraoperative TEE confirmed the absence of thrombus within LAAA, the surgical resection was approached via left lateral sternotomy with cardiopulmonary bypass (Fig. 7A and B). A huge LAAA (8.7 × 7 cm) was visualized with intact pericardium. Aneurysm neck was clamped and the aneurysm was excised (Fig. 7C and D). The pathological diagnosis proved to be a giant LAAA. The muscle fibers did not have morphological anomalies, although there was thinning of the myocardium due to fatty infiltration (Fig. 7E and F).

### 2.4 Follow-up

The postoperative course was uneventful. On postoperative day 5, TTE confirmed the disappearance of the LAAA from the left parasternal short-axis view of the aortic root. The patient was discharged on the seventh postoperative day. At his 1-month and 3-month follow-up visits, he remained asymptomatic without any adverse events.

## 3 Discussion

We reviewed PubMed from 1962 to 2016 to find relevant LAAA cases using keywords “left atrial appendage” and “aneurysm”. The search was limited to human studies in English language. Overall, 89 case reports and 6 case series of LAAA were identified.

### 3.1 Epidemiology and pathology

Parmley^[1]^ first reported LAAA in 1962.^[1]^ It can occur among patients of all the age groups, with a mean age 30 ± 20 years (range: fetus at 28 weeks to 88 years), although most patients (24.8%, 25/101) are in their third decade. This is probably because of progressive enlargement of the aneurysm with age. Although previous studies reported that there was no gender difference in the prevalence of LAAA, the percentage of female (53/101, 52.5%) patients seems to be slightly higher than that of male (45/101, 44.6%) patients. This is consistent with a previous report by Aryal et al^[2]^ that included 82 patients.

LAAA has been classified as congenital or acquired,^[3]^ with ninety percent of the cases being congenital. The reason is not clear, although the anomaly is probably due to congenital dysplasia of the atrial pectinate muscles.^[4]^ Acquired LAAA is often secondary to mitral valve disease, or other conditions leading to elevated left atrial pressure.^[5–7]^ The common histopathological finding of both congenital and acquired LAAA is fibrosis of the endocardium or myocardium. Hypertrophied myocardium with an increase in interstitial fibrous tissue may be seen in some cases.^[8,9]^

Rarely, LAAA has been reported to be associated with congenital cardiac abnormalities,^[7,10–13]^ including 5 patients with atrial septal defect, 1 patient with ventricular septal defect, 1 with anomalous pulmonary venous drainage, 1 with mitral valve cleft, and 1 with tricuspid atresia.

### 3.2 Clinical symptoms of LAAA

The symptoms associated with LAAA often occur during the second to the fourth decades of life. Thirty-four percent (34/101) of patients are asymptomatic and are diagnosed incidentally. On the other hand, among patients with symptoms, the main complaint is palpitation in 45 cases of the patients (44.6%), followed by dyspnea on exertion (28.7%, 29/101) and chest pain (11.9%, 12/101). When the aneurysms reach a larger size, they probably compress the left coronary artery or any of its divisions, leading to myocardial ischemia and atrial arrhythmia. Therefore, patients may experience palpitations, dyspnea, and chest pain. Palpitations on ECG proved to be atrial arrhythmia or supraventricular tachycardia. Electrocardiographically signs of atrial fibrillation/flutter were seen in 27 (26.7%) patients, supraventricular tachycardia in 10 (9.9%) patients, and sinus rhythm in 60 (59.4%) patients. A plausible explanation for the occurrence of supraventricular arrhythmias is the LAAA increases tension on the conduction system or a congenital defect in the conduction tissues exists.

Thromboembolic events may also present during the progression of LAAA. The dilatation of LAAA results in the stasis of blood, therefore increasing the risks of thrombi formation and systemic thromboembolism. In our study, 6 patients (5.9%) had systemic thromboembolism events.

## 4 Diagnosis

Methods used to diagnose LAAA include a chest x-ray, echocardiography, CT, and MRI. A standard chest x-ray was done in 88 (85.4%) patients. Although the findings were abnormal in all, they were nonspecific and might be interpreted as pericardial cyst, cardiac tumor, or mediastinal mass.

Echocardiography is considered to be the primary method of diagnosis. It shows a large saccular structure associated with the left atrium. Color Doppler echocardiography can also confirm an exchange of blood between the aneurysm and the left atrium. Eighty-six patients underwent echocardiography. TTE diagnosed LAAA accurately in only 24% (21/86) patients due to limited echo window. Although TTE has a low sensitivity for detection LAAA, it is useful to evaluate left ventricular function, abnormal myocardial movement, and valve regurgitation caused by the compression of LAAA. Besides, it helps to exclude other cardiac abnormalities.

TEE provides clear visualization of the structures surrounding atrium, making it effective for the diagnosis of LAAA. According to our data, TEE identified 30 cases with LAAA among 36 patients who underwent TEE. In the remaining 6 patients, the finding was reported either as enlarged left atrium or pericardial cryst. Due to its precise delineation, TEE should be mandatory if the diagnosis is ambiguous after evaluation by TTE.^[14]^

Other imaging modalities, including CT and MRI are useful for diagnosing LAAA as well as ruling out other differential diagnoses. MRI has the highest temporal resolution, making it the optimum approach for assessing the surrounding structures and cardiac anomalies. Ninety-one percent of patients (31/34) with LAAA were identified by cardiac MRI. However, MRI does have certain drawbacks, such as requiring a regular heart rhythm and exposing the patient to nephrotoxic contrast agents. Cardiac CT helps to evaluate the anatomy of coronary artery if compression of left coronary artery or its divisions is suspected. Twenty-seven patients underwent CT scan, and LAAA was detected in 24 cases. However, CT cannot provide functional data as accurately as echocardiography or MRI.

### 4.1 Management of LAAA

Surgical treatment is often recommended even in asymptomatic patients, as it can prevent the potential thromboembolic complications and treat associated atrial arrhythmias. A variety of surgical approaches for surgical resections have been reported.

Median sternotomy aided with cardiopulmonary bypass is the most commonly reported operative approach. This approach has been recommended in patients with intracardiac thrombus, for aortic clamping in this technique prevents systemic embolization during the resection of aneurysm. This approach is suitable for removal of large aneurysms with associated thrombi, but it comes at the cost of increasing invasiveness. Resection through a left lateral thoracotomy has been reported. Compared with the median sternotomy, this technique provides a better visual field and reduces the degree of invasiveness, although this is offset by an increase in procedural operator difficulty.

Seventy-four patients reported in the literatures were surgically treated. Eighty-five percent (63/74) of patients underwent median sternotomy. The size of the aneurysm was highly varied, from 4 × 3  to 22 × 15 cm. The average size of LAAA was 11 ± 5 × 7 ± 3 cm. Thrombi were diagnosed in 17 patients. In contrast, 10% of patients (11/101) without thrombi received the left lateral thoracotomy, with smaller LAAA size (the average size 7 ± 3 × 4 ± 2 cm). The median sternotomy is considered safer, especially in giant LAAA with intra-aneurysmal thrombi, whereas the left lateral thoracotomy is an option for patients without intra-aneurysmal thrombi seeking for less invasive approach. Both above surgical approaches are considered safe, as no patient died postoperatively. In fact, the prognosis is favorable, for freedom from recurrent symptoms and arrhythmia has been reported from 10 days to 8 years follow-up.^[15–17]^ Minimal endoscopic techniques have been less frequently described,^[18–20]^ although the outcomes have also been good.

A nonsurgical approach has been reported in 11 patients. The reason included denial of surgery with sinus rhythm (n = 5),^[21–25]^ right femoral artery embolism,^[26]^ the failure of addressing the occlusion of the right coronary artery in a patient with acquired LAAA,^[27]^ Eisenmenger syndrome in a patient with atrial and ventricular septal defect,^[7]^ older age (68 and 76 years, respectively) without intra-aneurysmal thrombus,^[11,28]^ and death secondary to massive cerebral embolism.^[3]^

## 5 Conclusion

In conclusion, LAAA is a rare cardiac anomaly. Echocardiography is considered the initial diagnostic tool, and cardiac CT as well as MRI helps to differentiate it from other abnormalities. The associated high risk of life-threatening complications and the relative ease of surgical removal suggest that prompt evaluation should be considered in patients with lesions adjacent to the left heart border.

## Supplementary Material

Supplemental Digital Content

## Supplementary Material

Supplemental Digital Content

## Supplementary Material

Supplemental Digital Content

## Supplementary Material

Supplemental Digital Content

## Supplementary Material

Supplemental Digital Content

## Supplementary Material

Supplemental Digital Content

## Supplementary Material

Supplemental Digital Content

## Supplementary Material

Supplemental Digital Content

## Supplementary Material

Supplemental Digital Content
